# Adherent Pericardium

**Published:** 1903-11-21

**Authors:** 


### ADHERENT PERICARDIUM.

For clinical purposes, Dr. Hale White [1] classifies cases of adherent pericardium into three groups, namely :—(1) those in which the condition is of no importance to the patient, (2) those in which it is of serious clinical importance owing to cardiac symptoms, and (3) those in which it is accompanied by universal adhesions affecting both pleuræ and the peritoneum (progressive hyaloserositis). The pericardium, Dr. Hale White states, does not owe its importance to the fact that it is a serous membrane. Its great value is, he declares, that it is a support to the heart, and prevents its over distension during muscular effort. It has been shown by Leonard

Hill and Barnard that if the pericardium be taken away it is possible to distend the heart much more than could be done while the pericardium was intact. When pericarditis occurs this fibrous sac becomes soft and pulpy so that it no longer forms a strong support to the heart. In addition to this there is the circumstance that pericarditis is often accompanied by myocarditis. Therefore, both the wall of the heart and the pericardium are weakened and the heart rapidly dilates in response to any increase of blood pressure, and if the heart dilates it must hypertrophy. The amount of work which the ventricle is called upon to perform increases very rapidly indeed as the radius of the curvature of the ventricle itself increases, so that a very little dilatation means a great deal more work for the heart, and consequently the heart hypertrophies. Hypertrophy therefore is a frequent result of pericarditis. With regard to the formation of adhesions there is one most important point, if they are of the tough variety and form when the heart is dilated they will hold out its walls and prevent the heart ever contracting up again. The treatment of a case of pericarditis must not be considered to have been successful simply because the temperature has fallen and the rub has disappeared. The index of success should be the absence of dilatation, and it is just that point which will decide whether a particular case shall be included in group (1) or group (2) mentioned above. If the heart is dilated when adhesions form, then the patient probably becomes a chronic invalid. If the organ is not dilated the presence of adhesions does not much matter. In the former case what happens is this: the heart being dilated, the auriculo-ventricular orifice does not close properly and mitral regurgitation results, and the sequelæ are the consequence of the mitral incompetence. So much is this the case that it is a good clinical maxim if a patient apparently suffering from mitral disease is not benefited by digitalis and rest in bed to infer that he may have adherent pericardium. To the rule that in the absence of dilatation pericardial adhesions do not much matter, Dr. Hale White makes an exception in the case of children, in whom the result is nearly always bad, probably because in them the inflammation is usually very acute and is accompanied by a good deal of myocarditis, and also because the heart is prevented from growing properly as time goes on.

The full recognition of the importance of preventing dilatation of the heart during an attack of pericarditis, and for some time afterwards is therefore the fundamental principle of treatment. With this in view the patient should be kept quiet in bed. He should be forbidden to make any muscular effort, because the attempt to do so would cause a rise in the blood pressure. Again the bowels should be kept loosely open so that there may be no straining during defæcation. Digitalis is valuable because it makes the heart contract up well, but care should be taken lest it makes the patient sick; if it has any tendency in that direction the drug should be given by the rectum. The value of sleep is great because it is accompanied by lowering of the blood pressure and slowing of the heart beats, and it is generally well therefore to give an injection of morphia at night. Alcohol, for obvious reasons, should be withheld.

[1] Guy's Hospital Gazette, Nov. 7.

